# Some Aspects of General Peritonitis

**Published:** 1908-04-11

**Authors:** 


### SOME ASPECTS OF GENERAL PERITONITIS.

General peritonitis has long been regarded as one of the most fatal of surgical conditions; in a sense this is a survival of one of the traditions of pre-historic times, pre-historic, that is, in the clinical sense of the word, for in years it is not so very long since death was the invariable result of this lesion.

Such abdominal troubles lay within the province of the physician, and although the phenomena of peritonitis were observed with great care, little could be done to cure the patient of his malady. Reliance was placed mainly on the administration of alcohol and other forms of stimulant, by the mouth, to increase his resistance and to help nature to overcome the trouble.

With the advent of antisepsis, it occurred to surgeons that it might be possible to do some good by opening the abdomen and at any rate investigating the condition of affairs. They had been previously deterred by their fear that any operation which involved opening the peritoneum was not only likely, but almost certain, to be followed by a state of affairs which would be worse than the original condition which they had sought to relieve. There could be no more striking proof of the reality of this terror on their part than is offered by the ingenuity with which abdominal operations were devised with the special object of avoiding the peritoneum. Ligature of the external iliac artery, for instance, which used to be more or less commonly performed for femoral aneurysm in the days gone by, was always done through a curved incision above Poupart's ligament, through which the peritoneum was deflected to the inner side and the vessel ligatured retroperitoneally. If suppuration followed, as it nearly always did, the peritoneum itself did not become involved in the inflammatory process, and no great harm was done. We suppose that nowadays no surgeon in his senses would attempt to perform this operation otherwise than transperitoneally.

In view of our present knowledge of acute inflammatory conditions, as they occur inside the peritoneum, it is, of course, difficult to understand the considerations which deterred our forefathers from opening the peritoneum. Even if we admit that acute appendicitis is a more common malady than it used to be (and there seems no reason to doubt that this is the case) they must have witnessed post-mortem examinations on cases where appendicitis had caused general peritonitis, and the death of the patient; and one wonders that it did not strike them that the condition could not possibly be made worse, and possibly might have been improved, by opening the abdomen and evacuating the abscess before it had had time to infect the whole cavity. They understood thoroughly the principles of treatment of inflammatory conditions in other parts of the body (who better?), but they had not the daring to apply those conditions to abdominal inflammation. Instead of that they folded their hands and said, "This is one of those dreadful cases of inflammation of the bowels, and the poor man must die."

However surprising it may seem, it was not until Lord Lister indicated the general principles of antisepsis that surgeons began to undertake coeliotomy in these hitherto heartrending cases; and it was then soon discovered that the peritoneum, instead of being an enemy, to interfere with whom was to court disaster, was in reality one of the best friends the surgeon could hope to come across. It was realised for the first time that if the actual cause of the inflammation was removed, this membrane would in many cases deal with and put an end to the results of the inflammation. As soon as experience began to show that the peritoneum was none the worse for surgical interference, thorough operations were performed as a routine in cases of generalised peritonitis. The focus was first sought for. If the appendix, it was removed after ligature of its base. If a perforated gastric ulcer, it was sewn up, to prevent any further leakage of stomach contents. And more than this, counter-incisions were made, and any free pus or sero-purulent material was removed by copious irrigations with hot saline solution, and drainage tubes were inserted in all the wounds.

This is almost universally the line of treatment adopted to-day, and by its means the mortality from general peritonitis has been reduced from almost 100 per cent. to something like 80.

But it has recently been shown, and we are indebted to an American surgeon for the discovery, that the vitality of the peritoneum is more amazing than we had thought. He has proved that irrigation at the operation is a waste of time in nearly all cases, because, however freely one irrigates, some pus is sure to be retained among the coils of intestine, and no mechanical means is sufficient to remove it thoroughly. Besides, in a very short time adhesions are formed round the tubes, so that drainage is not efficient after that time. He therefore advocates that no more time should be spent at the operation than is absolutely essential for dealing with the primary focus. As soon as this is done a small median suprapubic incision should be made and a tube put down to the pelvis. A tube should be put in both wounds. As soon as the patient comes round from the anæsthetic, he should be propped up in bed. If he suffers much pain from this morphia may be given; but it will be found generally that no objection is made to the position. And, finally (and this is the essential part of the treatment), saline solution at a temperature of from 105° F. to 110° F. must be injected into the rectum continuously. This can be done by a rectal tube inserted just within the sphincter, which is attached to an ordinary irrigator, the fluid being allowed to run in quite slowly. The management of the case is, of course, rather troublesome, and needs a nurse in constant attendance. But the result more than repays for this, for it is claimed that if the patient will only retain the saline, the chances are in favour of his recovery, and that by this means the mortality of general peritonitis will be enormously diminished.

(*To be continued.*)

